# Advanced glycation end product-modified low-density lipoprotein promotes pro-osteogenic reprogramming via RAGE/NF-κB pathway and exaggerates aortic valve calcification in hamsters

**DOI:** 10.1186/s10020-024-00833-8

**Published:** 2024-06-05

**Authors:** Xi Yang, Jingxin Zeng, Kaiji Xie, Shuwen Su, Yuyang Guo, Hao Zhang, Jun Chen, Zhuang Ma, Zezhou Xiao, Peng Zhu, Shaoyi Zheng, Dingli Xu, Qingchun Zeng

**Affiliations:** 1 State Key Laboratory for Organ Failure Research, Department of Cardiology, Nanfang Hospital, Southern Medical University, 1838 Northern Guangzhou Ave, Guangzhou, 510515 China; 2 Guangdong Provincial Key Laboratory of Shock and Microcirculation, Southern Medical University, Guangzhou, 510515 China; 3 Bioland Laboratory (Guangzhou Regenerative Medicine and Health Guangdong Laboratory), Guangzhou, 510005 China; 4 Department of Cardiovascular Surgery, Nanfang Hospital, Southern Medical University, Guangzhou, 510515 China

**Keywords:** AGE-LDL, CAVD, Inflammatory, Osteogenic, Hamsters, IL-37

## Abstract

**Background:**

Advanced glycation end product-modified low-density lipoprotein (AGE-LDL) is related to inflammation and the development of atherosclerosis. Additionally, it has been demonstrated that receptor for advanced glycation end products (RAGE) has a role in the condition known as calcific aortic valve disease (CAVD). Here, we hypothesized that the AGE-LDL/RAGE axis could also be involved in the pathophysiological mechanism of CAVD.

**Methods:**

Human aortic valve interstitial cells (HAVICs) were stimulated with AGE-LDL following pre-treatment with or without interleukin 37 (IL-37). Low-density lipoprotein receptor deletion (*Ldlr*^*−/−*^) hamsters were randomly allocated to chow diet (CD) group and high carbohydrate and high fat diet (HCHFD) group.

**Results:**

AGE-LDL levels were significantly elevated in patients with CAVD and in a hamster model of aortic valve calcification. Our in vitro data further demonstrated that AGE-LDL augmented the expression of intercellular cell adhesion molecule-1 (ICAM-1), interleukin-6 (IL-6) and alkaline phosphatase (ALP) in a dose-dependent manner through NF-κB activation, which was attenuated by nuclear factor kappa-B (NF-κB) inhibitor Bay11-7082. The expression of RAGE was augmented in calcified aortic valves, and knockdown of RAGE in HAVICs attenuated the AGE-LDL-induced inflammatory and osteogenic responses as well as NF-κB activation. IL-37 suppressed inflammatory and osteogenic responses and NF-κB activation in HAVICs. The *vivo* experiment also demonstrate that supplementation with IL-37 inhibited valvular inflammatory response and thereby suppressed valvular osteogenic activities.

**Conclusions:**

AGE-LDL promoted inflammatory responses and osteogenic differentiation through RAGE/NF-κB pathway in vitro and aortic valve lesions in vivo. IL-37 suppressed the AGE-LDL-induced inflammatory and osteogenic responses in vitro and attenuated aortic valve lesions in a hamster model of CAVD.

**Graphic Abstract:**

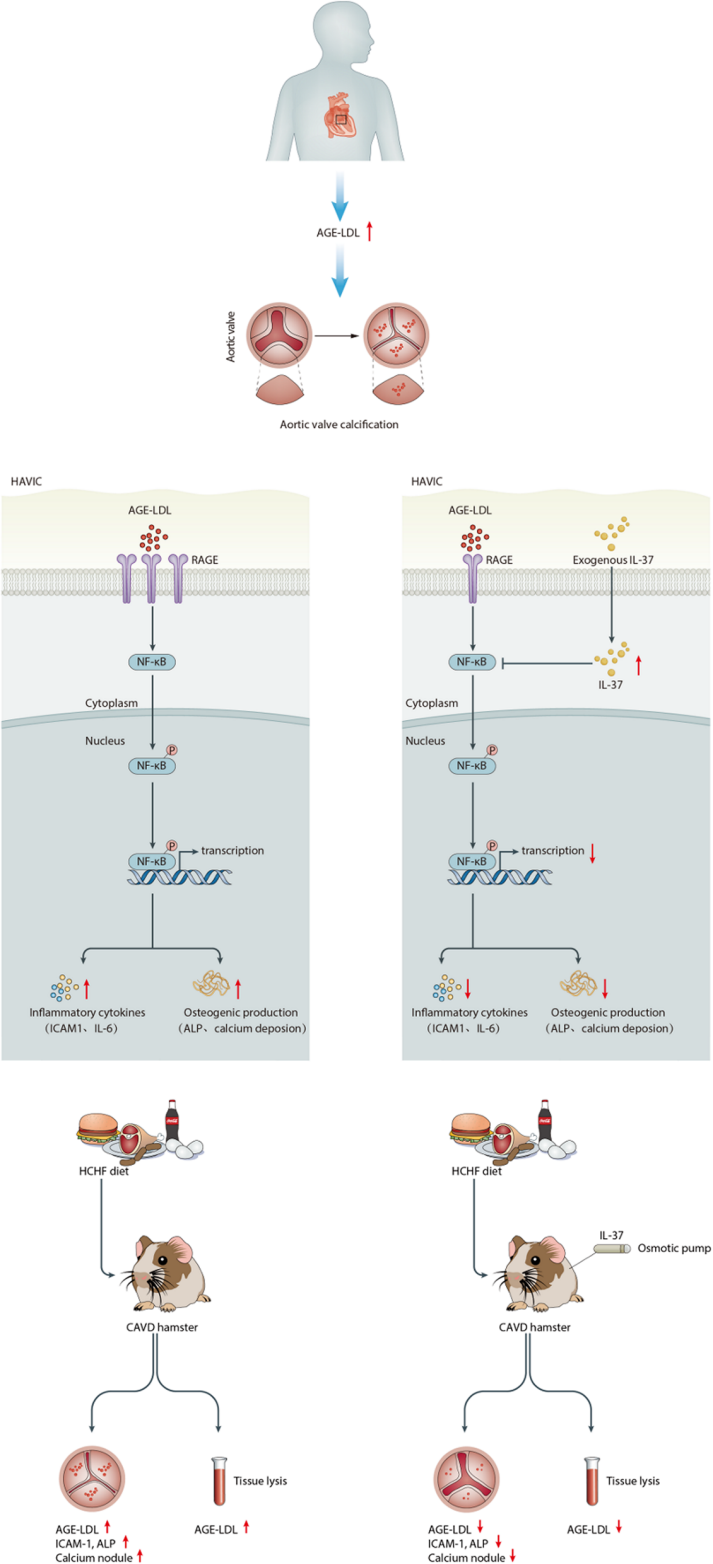

**Supplementary Information:**

The online version contains supplementary material available at 10.1186/s10020-024-00833-8.

## Introduction

Aortic valve stenosis (AVS) is mostly caused by calcific aortic valve disease (CAVD), which is fueled by ongoing inflammation (Otto and Prendergast 2014; Yutzey et al. 2014), and up to 25% of adults over 65 have valvular sclerosis (Small et al. 2017). The only current therapeutic options for symptomatic AVS are rigorous AVS monitoring and timing of aortic valve replacement. There are no medications available to prevent or treat CAVD, and not everyone is a candidate for aortic valve replacement. AVS had been considered as a degenerative disorder and characterized by calcium deposition on the aortic valve leaflets. Currently, histopathologic and clinical data suggest that calcific aortic valve disease is an active and multidimensional disorder that includes inflammation and calcium deposition rather than a simple degenerative process in which calcium accumulates on the valve cusps (Xu et al. 2020).

The accumulation of advanced glycation end products (AGEs) is attributed to the non-enzymatic glycation of proteins and lipids in hyperglycemic conditions (Lautenslager et al. 2011). AGE-LDL triggers inflammation via the TLR2/4-MyD88-dependent pathway and accelerates atherosclerosis by promoting lipid accumulation and a proinflammatory state (Cheng et al. 2013; Toma et al. 2009; Lopes-Virella and Virella 2003). However, the influence of AGE-LDL on valvular inflammation associated with CAVD progression was not evaluated.

Previous studies have shown that hyperlipidemia induce golden Syrian hamsters to develop atherosclerotic lesions of aortic valves, and that AGE proteins and LDL are deposited in the intima of atherosclerotic arteries (Sima et al. 1997). Hamsters with low-density lipoprotein receptor (*Lldr*) deletion (*Ldlr*^*−/−*^) hamsters exhibit impaired clearance of circulating large lipoproteins and accelerated progression of atherosclerosis (Reaves et al. 2000). However, its potential to induce aortic valve calcification in *Ldlr*^*−/−*^ hamsters requires further investigation.

The anti-inflammatory property of IL-37 was recognized by reduced cytokine expression and signaling kinase activity following challenge of inflammation. (Nold-Petry et al. 2015; Bulau et al. 2014; Sharma et al. 2008) Previous studies have demonstrated that the expression of IL-37 in mice attenuates aortic valve thickening following a prolonged exposure to endotoxin or high fat diet (Zeng et al. 2017). Despite considerable advances, whether IL-37 can regulate AGE-LDL mediated inflammation and osteogenesis is unclear. Blocking the AGE-LDL/RAGE axis by IL-37 may be a new therapeutic target for CAVD.

The present study examined the relationship between AGE-LDL and augmented osteogenic response, elucidated the mechanism of AGE-LDL in valvular inflammation and calcification, and determined the role of AGE-LDL as a risk factor for aortic valve calcification. We developed valvular calcification in hamsters fed a HCHF diet, examined the relationship between elevated AGE-LDL and hamster valvular calcification, and tested the hypotheses that the impact of IL-37 on valvular calcification was due to the regulation of AGE-LDL in hamsters.

## Materials and methods

All data, analytic methods, and study materials data are available within the article.

### Materials

Antibodies against IL-6 (ab9324, Mouse mAb), ALP (ab108337, Rabbit mAb) and β-actin (ab8226, Mouse mAb) were purchased from Abcam (San Francisco, CA, USA). Antibodies against IL-37 (PA5-115,405, Mouse pAb) were purchased from Invitrogen (Waltham, MA, USA). Antibodies against phosphorylated NF-κB (3033, Rabbit mAb), and total NF-κB (8242, Rabbit mAb) were purchased from Cell Signaling Technology (Beverly, MA, USA). Antibodies against H3k27me3 (A2363, Rabbit pAb), Histone3 (A2348, Rabbit pAb), ICAM-1 (A5597, Rabbit pAb) and AGE-LDL (customed, Rabbit Ab) were purchased from ABclonal (Wuhan, China). Specific small interfering RNAs (siRNAs) targeting RAGE were purchased from Ribobio, China. ALZET mini osmotic pumps were purchased from DURECT. Lipofectamine 3000 was purchased from Invitrogen (Carlsbad, CA, USA). Opti-MEM and Medium199 were purchased from Gibco (Carlsbad, CA, USA). IL-37 protein (1975-IL-025) was purchased from R&D System (Minneapolis, MN). Other chemicals and reagents were purchased from Sigma-Aldrich (St Louis, MO, USA).

### Human subjects

The Ethics Committee of Nanfang Hospital, P. R. China approved this study. From September 2018 to October 2019, individuals who entered the Department of Cardiology at Nanfang Hospital of Southern Medical University were recruited as patients with or without CAVD according to echocardiography. Patients with missing clinical data, acute coronary syndrome or stroke, severe kidney dysfunction (estimated glomerular filtration rate < 15 mL/min per 1.73 m^2^), hepatic dysfunction (AST ≥ 109 U/l and ALT ≥ 97 U/l), inflammatory bowel diseases, autoimmune diseases, congenital form (bicuspid), rheumatic heart disease, cancer, a history of chest radiation or chemotherapy and infections that had been treated with antibiotics or probiotics within 1 month were all excluded from the study. CAVD was characterized as enhanced echogenicity (aortic root echogenicity as control) and leaflet thickness (**≥ **3 mm) with peak AV velocity (**≥ **1.5 m/s), and there was increased echogenicity (aortic root echogenicity as control) (Li et al. 2022; Sengelov et al. 2018). All enrolled patients provided written informed consent before collection of a blood sample.

This study was approved by the Ethical Committee of Nanfang Hospital, P. R. China. Noncalcified aortic valves were collected from six patients (three males and three females with a mean age of 60.0 ± 2.2 years) who underwent aortic valve replacement due to acute aortic dissections at Nanfang Hospital. Calcified aortic valves were collected from six patients (three males and three females with a mean age of 71.0 ± 2.1 years) undergoing aortic valve replacement due to severe aortic valve stenosis at Nanfang Hospital. All patients gave informed consent for the use of their aortic valves for this study. The study was performed in accordance with the Declaration of Helsinki and approved by Nanfang Hospital.

### Cell isolation and culture

A previously published procedure was modified in order to isolate and cultivate AVICs (The et al. 2022). Briefly, aortic valve tissue was digested sequentially with collagenase, and the digest was then collected and centrifuged. For the purposes of the experiments and analyses that followed, cells were grown in M199 growth medium containing 10% fetal bovine serum, penicillin G, streptomycin, and amphotericin B. Nearly 90% of myofibroblast-like cells were clear after the third passage, at which point the cells were subcultured on plates and treated. For this investigation, cells from passages 4 through 6 were employed. The cells were grown in M199 growth medium containing penicillin G.

We have previously stated that endothelium contamination is not present in AVIC isolations or culture (Meng et al. 2008). We confirmed that all cultures utilized in the current investigation stained negatively for the von Willebrand factor. Vimentin is also expressed by every cell, and about 75% of cells are myofibroblasts, which also express alpha-smooth muscle actin.

### Cell treatment

To determine the effect of AGE-LDL on the expression of ALP, ICAM-1 and IL-6, HAVICs were treated with AGE-LDL (20, 50, 100 and 200 μg/ml) for 48 h. To determine the effect of RAGE in the AGE-LDL induced inflammatory and osteogenic responses, cells were treated with RAGE siRNA (60 nM) prior to stimulation with AGE-LDL (100 μg/ml). After stimulating cells with AGE-LDL (100 μg/ml) for 2-24 h, the impact of AGE-LDL on the phosphorylation of NF-B was confirmed. An IKK inhibitor (Bay11-7082; 5 μM) was added to the culture media 60 min before the addition of AGE-LDL to assess the function of NF-κB in the effect of AGE-LDL in HAVICs. Cells were pre-treated with IL-37 (1.0 ng/ml) before being stimulated with AGE-LDL (100 μg/ml) to ascertain its impact on the inflammatory and osteogenic responses caused by AGE-LDL.

### AGE-LDL preparation

LDL was purchased from Yiyuan Biotech, Inc. (Guangzhou, China). AGE-LDL was prepared according to established protocols. (Sima et al. 2010) AGE-LDL was made by incubating LDL (2 mg/ml) with D (+) glucose (0.2 M final concentration) for 4 weeks at 37 °C in sterile circumstances with antioxidants (1 mg/ml EDTA and 10 μM BHT).

Prepared AGE-LDL was treated with DNase/RNase and dialyzed in PBS (pH 7.4) under sterile conditions and stored at 4 °C for subsequent experiments. An amebocyte lysate test kit was used to detect the endotoxins in the preparation (Sigma, St. Louis, MO, USA). Trinitrobenzene sulfonic acid assay was used to assess the free, nonglycated amino groups. (Nivoit et al. 2003) To detect the extent of LDL protein glycation, the formation of pentosidine was measured by spectrofluorimetry (excitation 335 nm, emission 385 nm) (Krishnamurti et al. 1997).

### Cell transfection of siRNAs

Small interfering RNAs (siRNAs) were purchased from RiboBio (Guangzhou, China). In accordance with the instructions provided by the manufacturer, Lipofectamine 2000 (Invitrogen) and Opti-MEM (Gibco) were used to transfect HAVICs (40–50% confluence) with 60 nM siRNA. After transfection for 6 h, cells were stimulated with AGE-LDL (100 μg/ml) for 24 h and proteins were extracted for subsequent experiments.

### Immunoblotting

Cells were lysed with RIPA lysis buffer (Beyotime, Shanghai, China) containing phosphatase and protease inhibitor cocktails on ice and the lysate extracted. Samples were placed onto polyacrylamide gels with 10% sodium dodecyl sulfate, and the proteins were then transferred to polyvinylidene fluoride membranes. The PVDF membranes were incubated in 5% BSA for 1 h at room temperature and then reacted with the corresponding primary antibody (ICAM-1, IL-6, ALP, AGE-LDL, H3k27me3, Histone3, phosphorylated NF-κB p65, total NF-κB p65, RAGE and β-actin) dilutions overnight. The membranes were washed 3 times with TBST and then incubated with peroxidase-conjugated secondary antibody for 1 h at room temperature. The membranes were then treated with ECL reagent after several washings (FUDE Biological Technology Co., Hangzhou, China). The intensity of the expressed protein bands was quantified by ImageJ/Fiji software (NIH, Bethesda, MD, United States).

### Real-time RT-PCR

Human aortic valve tissues were used to isolate total RNA. To purify the total RNA, we fully reacted the homogenate with TRIzol reagent (Invitrogen). Reverse transcriptase (Takara Biotechnology, Dalian, China) was then used to turn the total RNA into cDNA using primer sequences for the desired molecules. Real-time quantitative PCR was then performed using a SYBR Green RT-PCR kit (Takara Biotechnology, Dalian, China) and detected using a LightCycler 480 II system (Roche Diagnostics, Basel, Switzerland). The 2^−ΔΔCT^ approach was used to quantify the relative mRNA levels of the target genes, with GAPDH serving as a normalization reference. The following primers were used to amplify specific cDNA fragments: human IL-37 (forward: 5′-CCC CAC CAT GAA TTT TGT TC-3′; reverse: 5′-CCT TTA GAG ACC CCC AGG AG-3′) (GenBank accession no. NM_0014439); GAPDH (forward: 5′-CAT GGC CTC CAA GGA GTA AG-3′; reverse: 5′-AGG GGT CTA CAT GGC AAC TG-3′).

### CHIP-qPCR

ChIP-qPCR was performed using a ChIP kit as described in the manufacturer’s instructions. Briefly, VICs were fixed using 1% formaldehyde, washed, and collected by centrifugation (1000*g* for 5 min at 4 °C). The pellet was resuspended in lysis buffer with 1% protease inhibitors and dithiothreitol, homogenized, incubated on ice for 10 min, and sonicated. The samples were centrifuged (13,000*g* for 10 min at 4 °C), and shared chromatin was used as input and incubated with an anti-H3K27me3 antibody. Rabbit IgG was used as isotype control. After precipitation using Pierce Protein A/G Magnetic Beads, followed by RNA and protein digestion, DNA was purified according to the manufacturer’s instructions. RT-qPCR was performed using SYBR GreenER qPCR SuperMix Universal (Vazyme, China). A detailed, step-by-step procedure is presented in Supplementary Methods. Forward primer: CATGGGGACTCCACTTGCAT, reverse primer: GATCAGGTGGCTTCCAGTCC.

### Aortic valve histology

The tissue of the aortic valve was rapidly embedded in an OCT with an optimal cutting temperature. The slicer temperature was pre-cooled down to − 20 ℃, then the heart sections were made parallel to the aortic valve orifice cross-section and sliced at 5 μm thickness. The frozen slices stored at − 20 ℃ for subsequent experiments. Aortic valve tissue can also be fixed in 4% paraformaldehyde fixative and soaked overnight to allow adequate fixation. The tissue is then dehydrated with gradients of ethanol and dewaxed with xylene, and the aortic valve tissue is placed in an embedding box. The cassettes were cooled rapidly on a − 20 ℃ freezing table and then sectioned at 5 µm thickness. Paraffin sections were stored at room temperature for subsequent experiments.

### Histological and immunohistochemical staining

We obtained 3-µm-thick sections of hamster and human aortic valves as in the previous step of the experiment, which were properly preserved and subsequently used for hematoxylin and eosin staining (H&E). Immunostaining was performed to identify ICAM-1, ALP, AGE-LDL, RAGE and IL-37 expression. First, the sections were dewaxed with xylene and graded ethanol and soaked in distilled water for 15 min. Antigen repair was carried out in the microwave using fresh sodium citrate buffer. Slices were first blocked in 10% goat serum, then exposed to the corresponding primary antibodies overnight, followed by secondary antibodies. Sections were fully reacted with DAB staining solution and stained with hematoxylin (Sigma-Aldrich). Image-Pro Plus (Media Cybernetics) was utilized to quantitate the results (positive staining area/total aortic valve area) of at least three aortic valve leaflets.

### Immunofluorescence staining

We used 4% paraformaldehyde as the fixative and 0.2% Triton X-100 as the fixatives. HAVICs were incubated in fixative for 15 min, followed by fixatives for 10 min. The cells were blocked with BSA for a further 30 min at room temperature and then treated overnight with a dilution containing primary antibody. The next day the cells were first rewarmed and then reacted with goat anti-rabbit secondary antibody (Santa Cruz Biotechnology Inc) for 60 min at room temperature. Finally, the cells were reacted with DAPI for 10 min at room temperature and washed thoroughly 3 times. Confocal images were acquired using a Leica.

### Alkaline phosphatase staining

Standard procedures were followed when staining with ALP. AP Staining Solution was added (Abcam, US) for 15 min after the cultured cells had been washed three times with PBS and fixed for two minutes with Fixing Solution. Staining was examined and photographed with an OLYMPUS IX83 microscope, densitometry analysis was performed using Image J.

### Alizarin Red S staining

The cells were stained for calcium salts according to the Alizarin Red S staining kit instructions. Firstly, cells were removed and washed three times at room temperature in pre-warmed PBS and then fixed in 70% ethanol for 20 min. Afterwards, the cells were reacted with alizarin red solution (LEA gene, Beijing, China) for 20 min at room temperature and then rinsed with distilled water until no excess dye was present. The staining was examined and recorded using an OLYMPUS IX83 microscope. For the next quantitative examination, cells were first washed with distilled water and then bleached with pre-prepared 10% acetic acid at 85 °C. The supernatant was taken and analyzed at 450 nm on a spectrophotometer.

### Animal models and treatment

Golden Syrian hamsters were originally purchased from Beijing Vital River Laboratory Animal Technology Co., Ltd. (Beijing, China). LDLR knock out (−/−) hamsters were generated by the CRISPR/Cas9 gene editing system and obtained from Hebei Ex&Invivo Biotechnology Co., Ltd. (Hebei, China). The hamsters were housed in a pathogen-free facility of Southern Medical University, under standard conditions of temperature and humidity with an alternating 12-h light/dark cycle. The study was carried out in compliance with the guidelines of the NIH’s eighth edition of the Guide for the Care and Use of Laboratory Animals, which was authorized by Southern Medical University’s ethics review board. After the acclimation period of 5–7 days, the first batch of hamsters were randomly assigned to groups fed a high carbohydrate and high fat (HCHFD: 58% of total calories are provided from fats, 25.5% of carbohydrate) diet and chow diet (CD: 20% protein and 4% of calories from fat). The second batch of hamsters were randomly assigned to groups treated with IL-37 and normal saline (NS) by osmotic pump one week before HCHFD. Infiltration of 20 ug of IL-37 suspended in 2 ml of normal saline. Food intake was monitored twice weekly. All diets were obtained from Guangdong Medical Lab Animal Center and hamsters were monitored for 16 weeks throughout that time.

In order to get the optimal image quality and views, the hamsters were anesthetized with 2% isoflurane (S.A. Abbott N.V., Ottignies, Belgium) and 2L O_2_/min as the carrier gas, and positioned in left lateral decubitus on a hardwood bench, as previously described (Lopez et al. 2012). Doppler ultrasound and M-mode echocardiography using high-resolution (3255 MHz) ultrasound (Visual Sonics Vevo 2100) was performed to evaluate aortic valve function. Hamsters were fasted for 18 h and blood was obtained for the measurement of AGE-LDL, triglycerides (TG), total cholesterol (TC), low-density lipoprotein cholesterol (LDL-C), AGE-LDL, IL-8, IL-6 and IL-1β levels. At the end of the study, the hamster was injected intraperitoneally with pre-dosed sodium pentobarbital. Normal saline was perfused from the right ventricle of the hamster to the whole heart to remove residual blood from the heart, which was removed and quickly placed in PBS for cleaning. The hearts were preserved in 4% paraformaldehyde after the fat and connective tissues were removed so that they could be stained with H&E, von Kossa, and immunohistochemistry.

### Evaluation of AGE-LDL levels

The serum was prepared by incubating whole blood at RT for 30 min to allow clotting before being centrifuged at 1880*g* for 10 min at RT. The serum was finally transferred to a new tube and centrifuged at 2500*g* for 10 min at RT and serum was stored at − 80 ℃ for subsequent testing.

Add AGE-LDL calibrator and samples to be tested in a microtiter plate pre-coated with antibodies against AGE-LDL. Then, add another antibody against glycated LDL labeled with Horseradish Peroxidase (HRP). Incubate and thoroughly wash. Add 0.01% hydrogen peroxide and 0.1% 3,3′,5,5′-tetramethylbenzidine (0.1% TMB) as the substrate. Under the catalysis of HRP, a blue-colored product is generated. Upon the action of the stop solution, the color converts to yellow. Absorbance of standards and samples were determined spectrophotometrically at 450 nm. Results were plotted against the linear portion of the standard curve and calculate the concentration of AGE-LDL in the sample.

### Masson staining

Masson staining was employed to detect the level of collagen in aortic valves using Masson staining Kits (Solarbio, G1340, CN), according to the manufacturer’s protocol. The fractional area of collagen fibrosis components (blue) in the aortic valve was acquired ImageJ 1.55 (National Institutes of Health).

### Statistical analysis

Statistical analyses were performed using Prism Software (GraphPad). Data are presented as mean ± SEM. Differences between groups were tested by Mann–Whitney U test as appropriate and t-test was applied to compare data between two groups. The categorical variables are presented as count (percent) and comparisons between groups were performed using χ^2^. Logistic regression analysis was performed to determine the odds ratio and 95% CI for CAVD. Statistical significance was defined as *P* ≤ 0.05.

## Results

### Patients with CAVD have increased levels of AGE-LDL

**Fig. 1:**
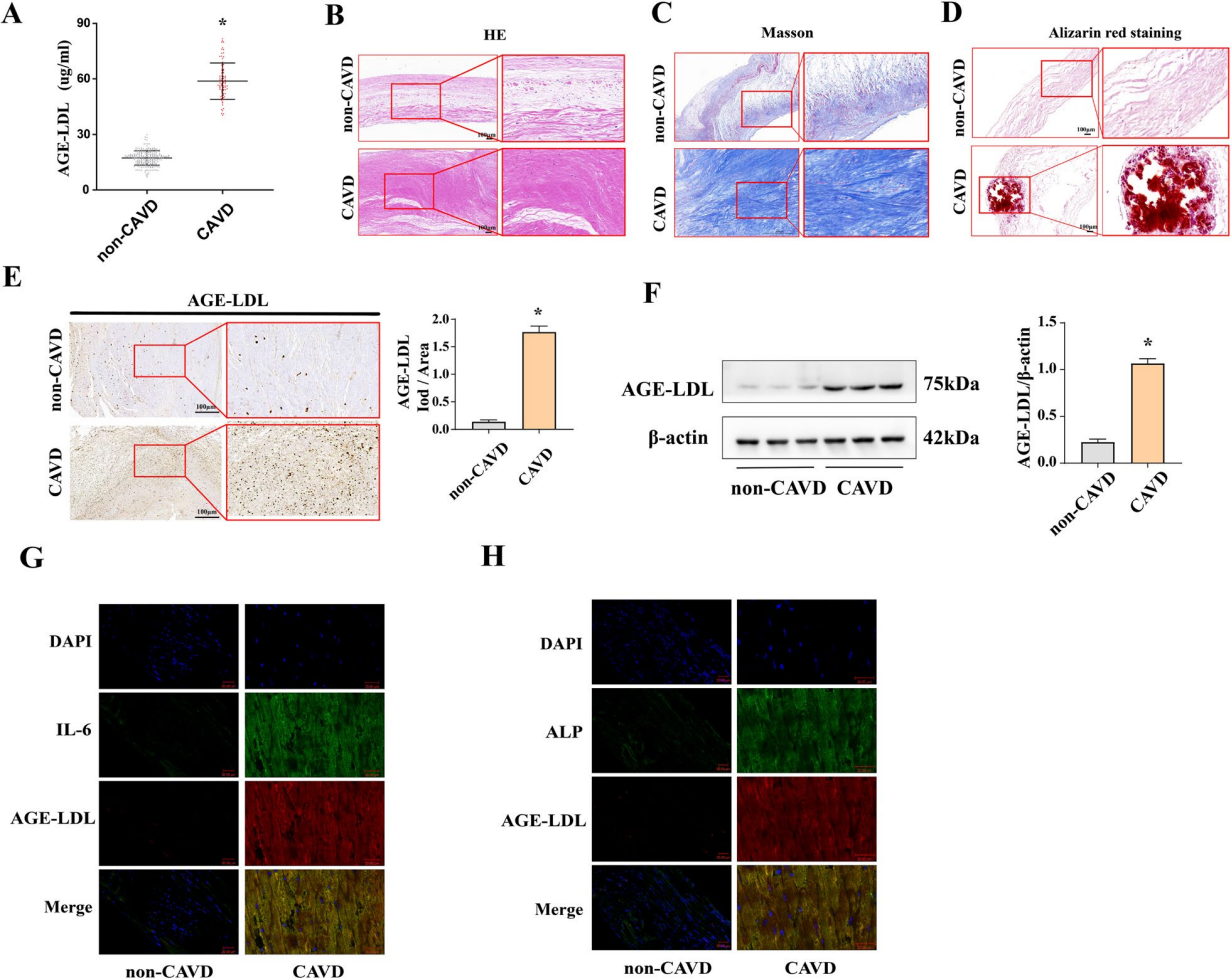
The association between AGE-LDL levels and aortic valve calcification. **A** Patients in the CAVD group (n = 275) had higher levels of AGE-LDL compared to the non-CAVD group (n = 81). Data are presented as means ± SD. **B** Representative histology images of aortic valve structure from calcified and non-calcified tissue stained with hematoxylin and eosin (HE) (bar = 100 µm). **C** Representative histology images of aortic valve structure from calcified and non-calcified tissue stained with Masson. **D** Representative histology images of aortic valve structure from calcified and non-calcified tissue stained with Alizarin Red S. **E** The semiquantitative analysis of immunohistochemical staining show that patients in the CAVD group had higher AGE-LDL levels compared to the non-CAVD group (bar = 100 µm). n = 5. **F** The semiquantitative analysis of immunoblots show that patients in the CAVD group had higher AGE-LDL protein levels compared to the non-CAVD group. **G** The representative immunofluorescence staining show that the co-localization area and range of AGE-LDL and IL-6. **H** The representative immunofluorescence staining show that the co-localization area and range of AGE-LDL and ALP, n = 5. *P < 0.05. vs. non-CAVD. Data are presented as means ± SEM. CAVD, calcified aortic valve disease; AGE-LDL, advanced glycation end product-modified low-density lipoprotein

We excluded patients who did not meet the inclusion criteria, had missing clinical data, or did not provide blood samples from the 582 participants who signed informed consent forms. Ultimately, we analyzed serum samples and clinical data from 356 participants. A detailed flow chart (Supplemental material Fig. S1) depicted participant selected from September 2018 to October 2019, individuals who entered the Department of Cardiology at Nanfang Hospital of Southern Medical University were recruited as patients with or without CAVD according to echocardiography. The characteristics of the valve and blood study are summarized in Supplementary material Tables S1 and S5, respectively. Patients in the CAVD group had higher levels of serum glucose and were older compared to non-CAVD patients. Compared to non-CAVD patients, those with CAVD were more likely to present with a history of diabetes, heart failure, and higher blood cholesterol. The other clinical factors did not significantly differ between the two groups. The cross-sectional comparison of serum AGE-LDL levels between the two groups of patients suggested that patients in the CAVD group had higher levels of AGE-LDL both in serum and tissues compared to the non-CAVD group (Fig. 1A). Further research revealed that AGE-LDL was a predictor of CAVD by univariate regression analysis. The association remained significant following age, sex, and race adjustments. The significant predictive value of AGE-LDL was preserved in model 2 and after adjustment for all factors including age, sex, LDL-C, TG, UA, CR, HbA1c, hypertension, diabetes mellitus, coronary artery disease and heart failure (Supplemental material Table S2). There was no statistical difference of AGE-LDL levels between DM and non-DM patients with CAVD (Supplemental material Table S3).

To further investigate the association between AGE-LDL and aortic valve calcification, we analyzed histological morphology of human aortic valve with/without CAVD. We observed that three layers previously mentioned were disorganized with obvious rearrangement in each layer (Fig. 1B). Figure 1C results revealed that collagen fibers were stained blue and myofibers were stained red. Figure 1D Results revealed that calcium deposits appeared as orange-red color, contrasting with the surrounding tissue by alizarin red staining. As shown in Fig. 1E, AGE-LDL protein levels was increased in tissues with calcification. Similarly, immunoblotting proved that the expression of AGE-LDL was increased in calcified aortic valves (Fig. 1F). These results suggest that valvular thickening is accompanied by increased AGE-LDL levels in the lesion area**.** The observation that AGE-LDL co-localizes with both IL-6 and ALP indicates that potential interactions or functional associations between AGE-LDL, proinflammation and calcification.

### The high carbohydrate and high fat diet promotes the aortic valve lesion formation in hamsters

**Fig. 2:**
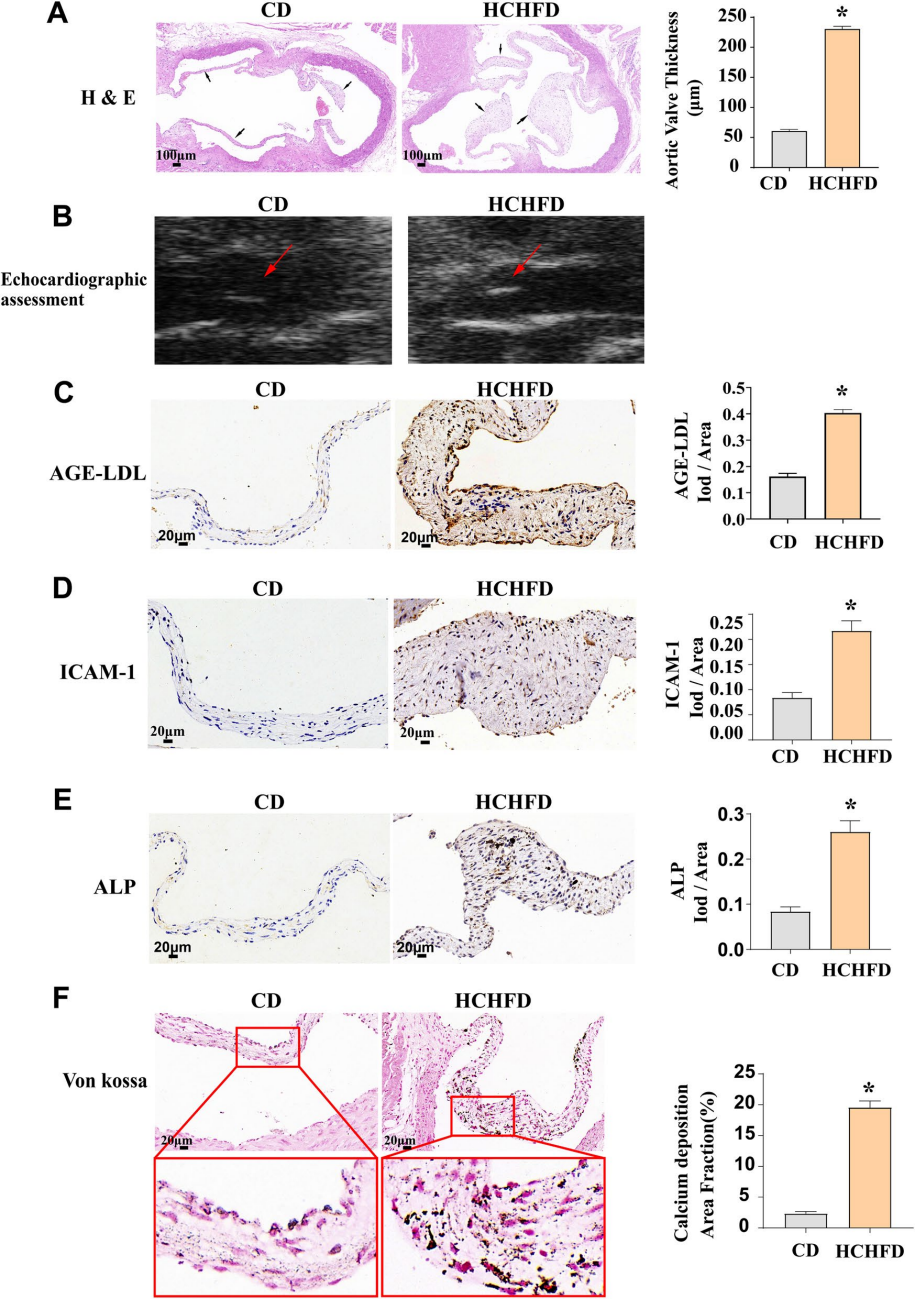
The HCHFD results in AGE-LDL accumulation and augmented inflammatory and osteogenic responses of aortic valve in *Ldlr*^*−/−*^ hamsters. **A** Histology images show that hamsters fed a HCHFD exhibit increased aortic leaflet thickening (bar = 100 µm). **B** Echocardiographic images of the leaflet thickness. **C** Immunohistochemistry images show that the expression of AGE-LDL in the aortic valve was mediated by a HCHFD. **D** Immunohistochemistry images show that a HCHFD increases the level of ICAM-1 in aortic valve. **E** Immunohistochemistry images show that a HCHFD increases the level of ALP in aortic valve. **F** Histology images show that a HCHFD increased calcium deposition in aortic valve (black calcium particles). (bar = 20 µm). CD group n = 9. HCHFD group n = 7. *P < 0.05. vs. CD group. The data are presented as the means ± SEM. CD, chow diet; HCHFD,  high carbohydrate and high fat diet; ICAM-1, intercellular cell adhesion molecule-1; ALP, alkaline phosphatase

The LDLR knock out (LDLR KO) hamsters fed HCHF diet exhibited a reduced aortic valve orifice area, higher levels of peak velocity, peak pressure, and AGE-LDL compared to the hamsters fed chow diet at the time of euthanasia (Supplemental material Fig. S2). We measured the aortic valve thickness and AGE-LDL levels in LDLR knock out (LDLR KO) hamsters given a CD and an HCHFD to determine the impact of the HCHFD diet on serum AGE-LDL and CAVD. The findings showed that LDLR KO hamsters fed an HCHFD had higher serum AGE-LDL levels and thicker aortic valves than LDLR KO hamsters fed a CD (Fig. 2A and B). In addition, the valve thickening was accompanied by elevated levels of AGE-LDL, ICAM-1 and ALP in the valvular tissue (Fig. 2C–E). We also observed that AGE-LDL promoted the accumulation of calcium deposits and the formation of calcification nodules in LDLR KO hamsters. These in *vivo* experiments demonstrated that a high carbohydrate and high fat diet enhanced the synthesis of AGE-LDL, thereby increasing aortic valve lesions.

### AGE-LDL induces the inflammatory and osteogenic responses in HAVICs

**Fig. 3:**
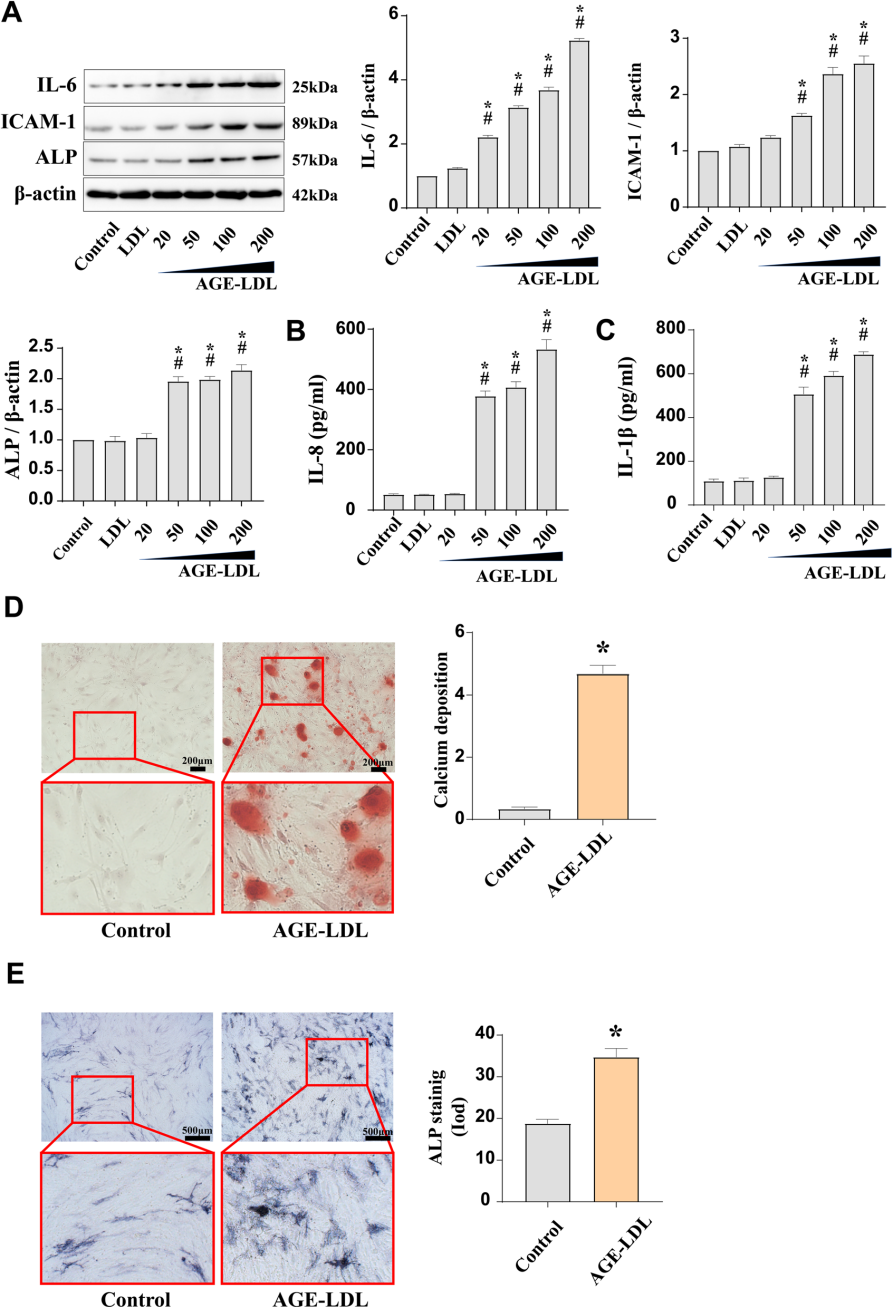
AGE-LDL upregulates the inflammatory and osteogenic responses in HAVICs. Stimulation HAVICs with LDL (100 µg/ml) and different concretions of AGE-LDL (20, 50, 100, 200 µg/ml) to analyze the inflammatory and osteogenic responses. **A** Immunoblots images and the semiquantitative analysis of ICAM-1, IL-6 and ALP expression in HAVICs. n = 5. **B** Analysis of IL-8 released from AGE-LDL stimulated cells into the supernatant by ELISA. n = 5. **C** Analysis of IL-1β released from AGE-LDL stimulated cells into the supernatant by ELISA. n = 5. **D** AGE-LDL (100 µg/ml) stimulated HAVICs for 21 days and Alizarin Red S staining showed a significant increase in calcium deposition. (sbar = 200 µm). n = 5. **E** AGE-LDL (100 µg/ml) stimulated HAVICs for 21 days and ALP staining showed a significant increase in ALP activity. (bar = 500 µm). n = 5. *P < 0.05. vs. untreated control; #*P* < 0.05 vs. LDL alone. The data are presented as the means ± SEM. IL-6 = interleukin-6; other abbreviations as in Fig. 1

We examined the impact of AGE-LDL at different concentrations on the inflammatory and osteogenic responses in HAVICs to further investigate the association between AGE-LDL and aortic valve calcification. As shown in Fig. 3A, ICAM-1, IL-6, and ALP protein levels increased in a dose-dependent manner in response to AGE-LDL stimulation. We also found that the groups challenged by AGE-LDL had higher levels of IL-8 and IL-1 in the supernatant (Fig. 3B and C). Additionally, AGE-LDL promoted the accumulation of calcium deposits and the development of calcification nodules in HAVIC cultures (Fig. 3D). AGE-LDL caused a higher increase in ALP activity compared to controls as presented in Fig. 3E. These results show that exposure to AGE-LDL promotes inflammatory response and osteogenic differentiation in HAVICs.

### Silencing of RAGE suppresses the inflammatory and osteogenic responses induced by AGE-LDL

**Fig. 4:**
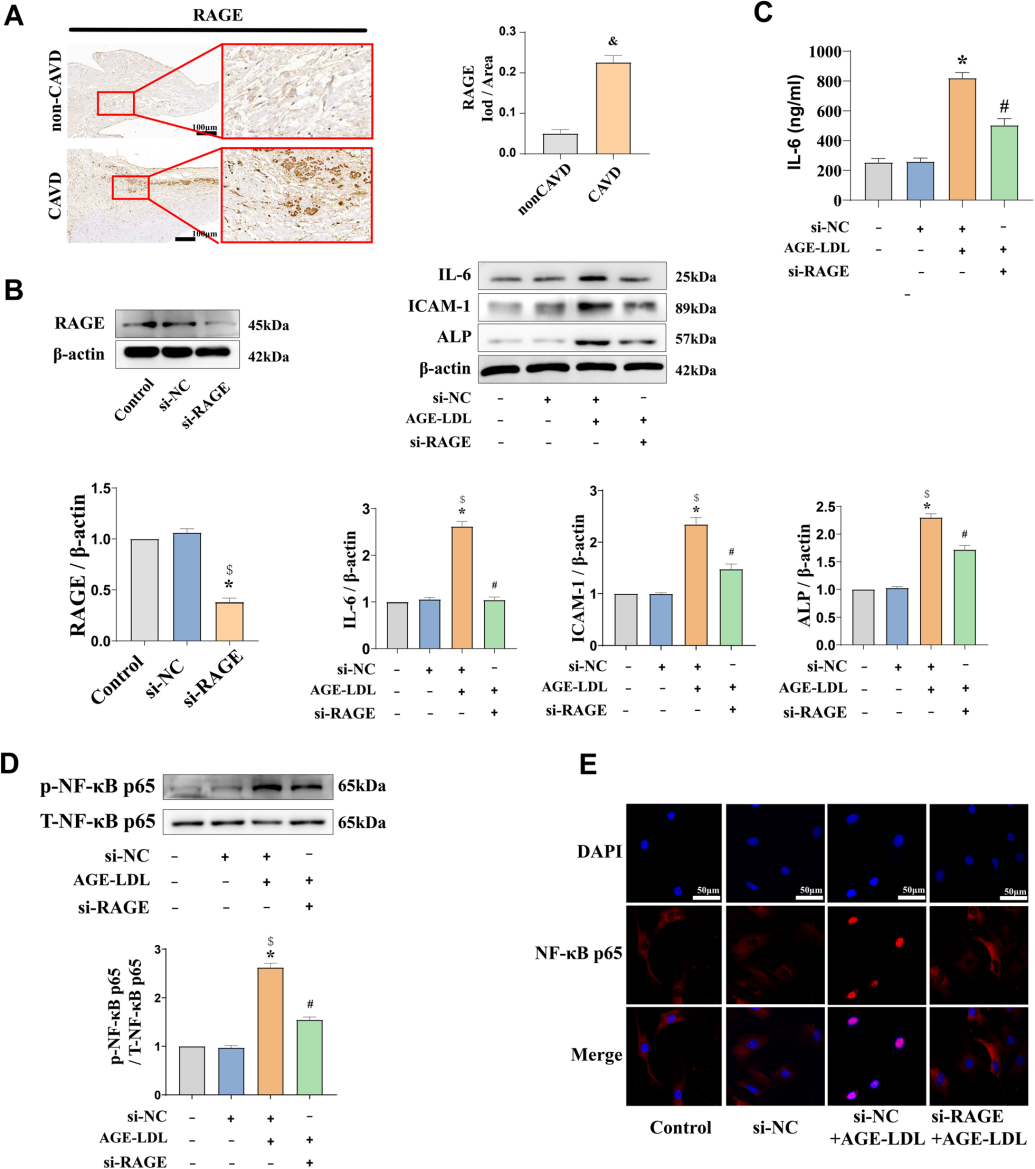
RAGE mediates the inflammatory and osteogenic responses as well as NF-κB p65 nuclear translocation induced by AGE-LDL. **A** Representative immunohistochemical image and semiquantitative analysis show that the levels of RAGE are higher in CAVD group than in the non-CAVD group (bar = 100 µm). **B** HAVICs were transfected with si-RAGE for 6 h prior to AGE-LDL treatment and immunoblotting analysis showed that the expression of ICAM-1, IL-6, ALP and NF-κB p65 was significantly inhibited. **C** ELISA showed that the significant increase IL-6 level induced by AGE-LDL treatment was attenuated by si-RAGE in culture media. **D** Representative images from immunofluorescence staining show that the silencing of RAGE reduces AGE-LDL induced NF-κB p65 translocation to nucleus. n = 5. ^&^*P* < 0.05. vs. non-CAVD. *P < 0.05. vs. untreated control, ^$^*P* < 0.05. vs. si-NC group, ^#^*P* < 0.05. vs. AGE-LDL + si-NC group alone. The data are presented as the means ± SEM. RAGE, Receptor for advanced glycation end product; si-NC, negative control small interfering RNA; other abbreviations as in Figs. 1–3

It has been reported that RAGE is a signal transduction receptor that binds to AGE-LDL in a range of cell types, including endothelial, smooth muscle, mesangial and mononuclear cells (Lopez et al. 2012; Sima et al. 2010). To determine the effect of RAGE in aortic valve calcification, we analyzed the protein levels of RAGE in aortic valve tissue from patients with or without CAVD. As shown in Fig. 4A, RAGE protein levels were increased in tissues with calcification. In the analysis of the effect of RAGE on inflammatory and osteogenic response, we found that silencing of RAGE markedly reduced the levels of ICAM-1, IL-6 and ALP triggered by AGE-LDL in HAVICs (Fig. 4B and C). Furthermore, to test whether RAGE involved in NF-κB p65 phosphorylation, we added si-RAGE prior to the addition of AGE-LDL to cells, as shown in Fig. 4D, we found pretreatment with si-RAGE attenuated the phosphorylation of NF-κB p65. Similarly, HAVICs pretreated with si-RAGE show that decreased AGE-LDL induced NF-κB p65 intranuclear translocation (Fig. 4E). Altogether, RAGE affects NF-κB p65 into the nucleus, which in turn affects the inflammatory and osteogenic response of HAVICs.

### NF-ĸB plays a mechanistic role underlying AGE-LDL induced inflammatory and osteogenic response in HAVICs

**Fig. 5:**
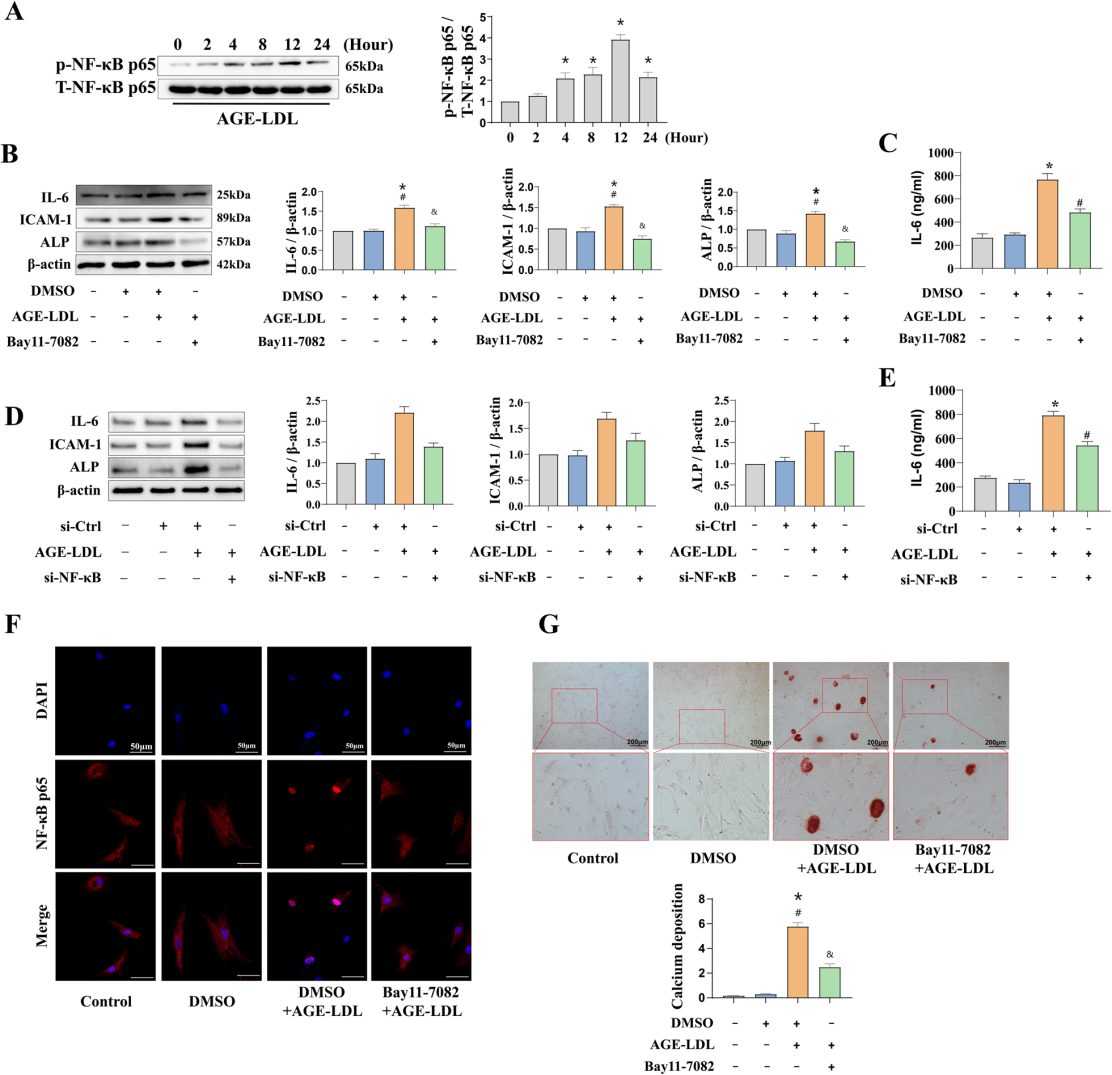
The NF-κB signaling is involved in mediating the AGE-LDL induced inflammatory and osteogenic responses in HAVICs. **A** HAVICs were treated with AGE-LDL for 0–24 h and the phosphorylation of NF-κB were detected by immunoblotting. **B** Pretreatment of HAVICs with Bay11-7082 prior to AGE-LDL stimulation showed a corresponding decrease in ICAM-1, IL-6 and ALP levels. **C** ELISA showed that the significant increase IL-6 level induced by AGE-LDL treatment was attenuated by Bay11-7082 in culture media. **D** Pretreatment of HAVICs with NF-κB si-RNA prior to AGE-LDL stimulation showed a corresponding decrease in ICAM-1, IL-6 and ALP levels. **E** ELISA showed that the significant increase IL-6 level induced by AGE-LDL treatment was attenuated by Bay11-7082 in culture media.** F** Pretreatment of HAVICs with Bay11-7082 prior to AGE-LDL stimulation showed that AGE-LDL-induced nuclear translocation of NF-κB p65 was abolished significantly inhibited. (bar = 50 μm). **G** Pretreatment of HAVICs with Bay11-7082 prior to AGE-LDL stimulation and stained by Alizarin red S staining showed that AGE-LDL-induced calcium deposit formation was significantly reduced. (bar = 200 µm). n = 3. *P < 0.05. vs. untreated control, ^#^*P* < 0.05. vs. DMSO group, ^&^*P* < 0.05. vs. DMSO + AGE-LDL. The data are presented as the means ± SEM

Previous studies have determined that the NF-ĸB pathway’s activation plays a significant role in the inflammatory and osteogenic response in HAVIC (Zhang et al. 2022; Zeng et al. 2012). We elucidated how AGE-LDL affected the phosphorylation of NF-κB p65 as a result. We discovered that AGE-LDL increased NF-κB p65 phosphorylation (Fig. 5A). We pretreated cells with IKK inhibitor (Bay11-7082) and si-NF-κB before AGE-LDL stimulation to further support the function of NF-κB in the modulation of inflammatory and osteogenic responses. Inhibiting or knocking down NF-κB with Bay11-7082 or NF-κB si-RNA in HAVICs prevented the AGE-LDL-induced elevation of ICAM-1, IL-6 and ALP (Fig. 5B–E). Similarly, HAVICs pretreated with Bay11-7082 showed that decreased AGE-LDL induced NF-κB p65 intranuclear translocation (Fig. 5F). AGE-LDL-induced calcium deposition was reduced by Bay11-7082 treatment (Fig. 5G). In present study, we demonstrated the mechanistic role of NF-κB phosphorylation in mediating AGE-LDL induced inflammatory and osteogenic response in HAVICs.

### IL-37 suppresses the inflammatory and osteogenic responses through inhibition of NF-κB

**Fig. 6:**
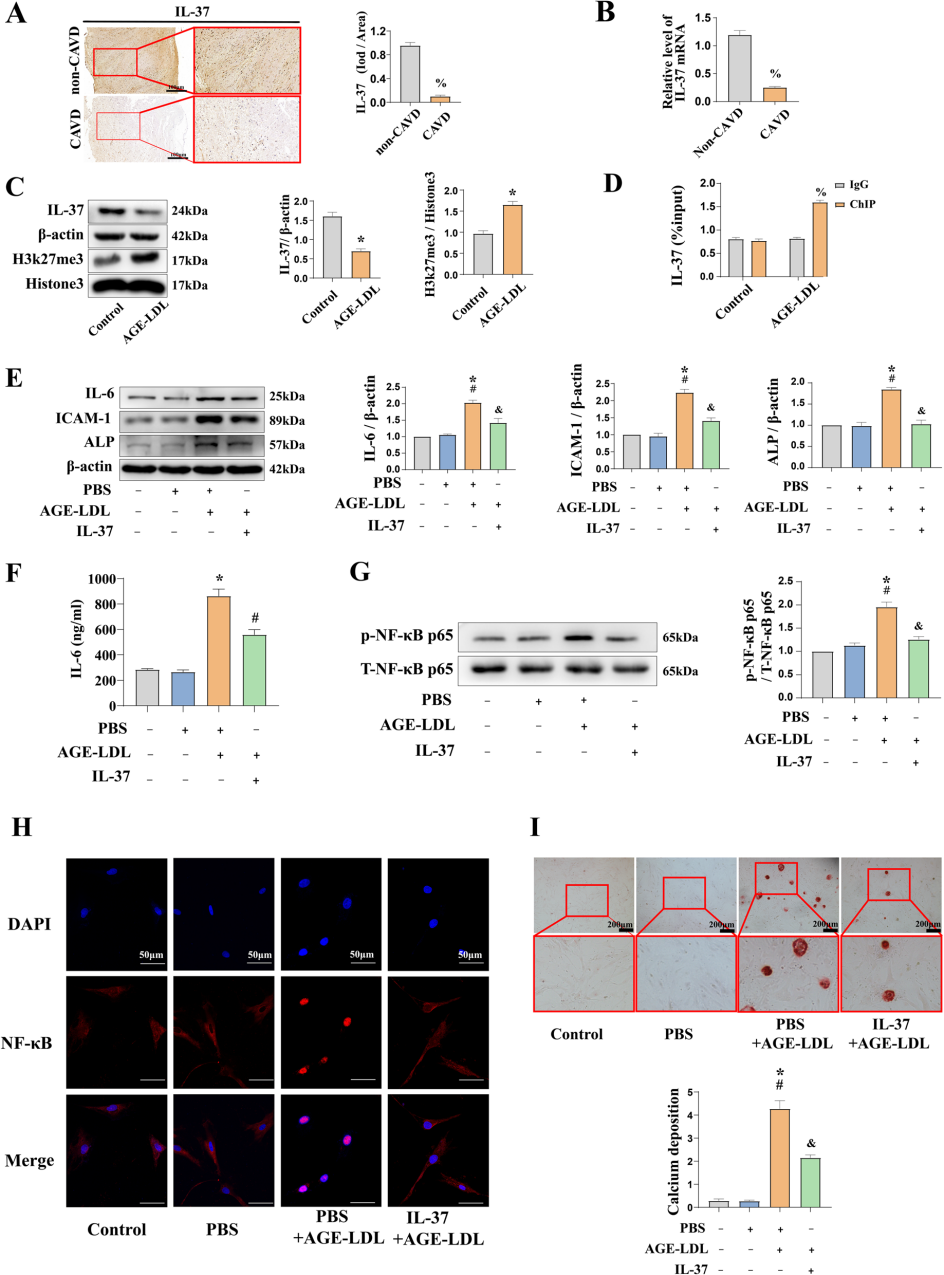
IL-37 suppressed the expression of inflammatory mediators by inhibiting the activation of NF-κB p65. **A** Semi-quantitative analysis of immunohistochemical images showed that patients in the non-CAVD group had higher levels of IL-37 in the aortic valve tissue compared to the CAVD group (bar = 100 µm). **B** PCR analysis show that human calcified aortic valve expresses lower levels of IL-37 mRNA. **C** Immunoblotting was used to detect the expression of H3k27me3 and IL-37 after treating HAVICs with AGE-LDL (100 μg/ml) for 48 h. **D** CHIP-qPCR analysis show that AGE-LDL treatment increases the enrichment of H3K27me3 in IL-37 promoter region. **E** IL-37 (1.0 ng/ml) treated HAVICs 1 h prior to AGE-LDL (100 μg/ml) treatment for 48 h. Immunoblotting analysis of ICAM-1, IL-6 and ALP levels. **F** ELISA showed that the significant increase IL-6 level induced by AGE-LDL treatment was attenuated by IL-37 in culture media. **G** AGE-LDL (100 μg/ml) treated HAVICs for 12 h in the presence or absence of IL-37 (1.0 ng/ml). Immunoblotting analysis shows an increase in phosphorylated NF-κB p65, indicating an elevation in NF-KB phosphorylation modifications. **H** Immunofluorescence images of NF-κB p65 nuclear translocation (bar = 50 μm). The immunofluorescence results indicate that NF-κB p65 translocates to the nucleus after stimulation with AGE-LDL in HAVICs, suggesting activation of the non-phosphorylated form of NF-κB p65.** I** Alizarin red S staining of calcium deposition (bar = 200 µm). n = 5. *P < 0.05. vs. untreated control, ^#^*P* < 0.05. vs. PBS, % *P* < 0.05. vs. IgG, ^&^*P* < 0.05. vs. PBS + AGE-LDL. The data are presented as the means ± SEM. IL-37 = interleukin-37; other abbreviations as in Figs. 1–3

Our previous studies found that IL-37, an anti-inflammatory factor, has protective effect against aortic valve calcification (Zeng et al. 2017). We also found that IL-37 level was notably lower in calcified aortic valve, compared with that in non-CAVD patients (Fig. 6A and B). Previous study reported that H3K27me3 expressed in macrophages in response to bacterial products and inflammatory cytokines (Santa et al. 2007). To investigate whether the decrease of IL-37 on CAVD was due to AGE-LDL, we analyzed the protein levels of IL-37 and H3K27me3 following AGE-LDL stimulation. We found that the expression of IL-37 was decreased and H3k27me3 was increased by the stimulation of AGE-LDL (Fig. 6C). The chromatin immunoprecipitation (CHIP)-qPCR results showed that AGE-LDL increased the binding of H3K27me3 to IL-37 promoter, which in turn inhibited the expression of endogenous IL-37 (Fig. 6D). Interestingly, pretreated cells with exogenous IL-37 prior to stimulation with AGE-LDL resulted in a reduction in the levels of ICAM-1, IL-6 and ALP in HAVICs (Fig. 6E and F). To elucidate the molecular mechanisms of IL-37 attenuates the inflammatory and osteogenic responses caused by AGE-LDL, we tested the effect of IL-37 on NF-κB signaling. We found that pretreatment with IL-37 suppressed AGE-LDL-induce phosphorylation of NF-κB (Fig. 6G). Similarly, HAVICs pretreated with IL-37 decreased AGE-LDL induced NF-κB p65 intranuclear translocation (Fig. 6H). To confirm the role of IL-37 in modulating osteogenic responses, we examined calcium deposition in HAVICs. We found that IL-37 reduced the formation of calcium deposits and calcification nodules in HAVICs following AGE-LDL stimulation (Fig. 6I). These findings indicate that IL-37 negatively modulates AVIC inflammatory and osteogenic responses and suppresses in vitro osteogenic activity through NF-κB signaling.

### IL-37 attenuates the formation of aortic valve lesions in hamsters administered by HCHFD

**Fig. 7:**
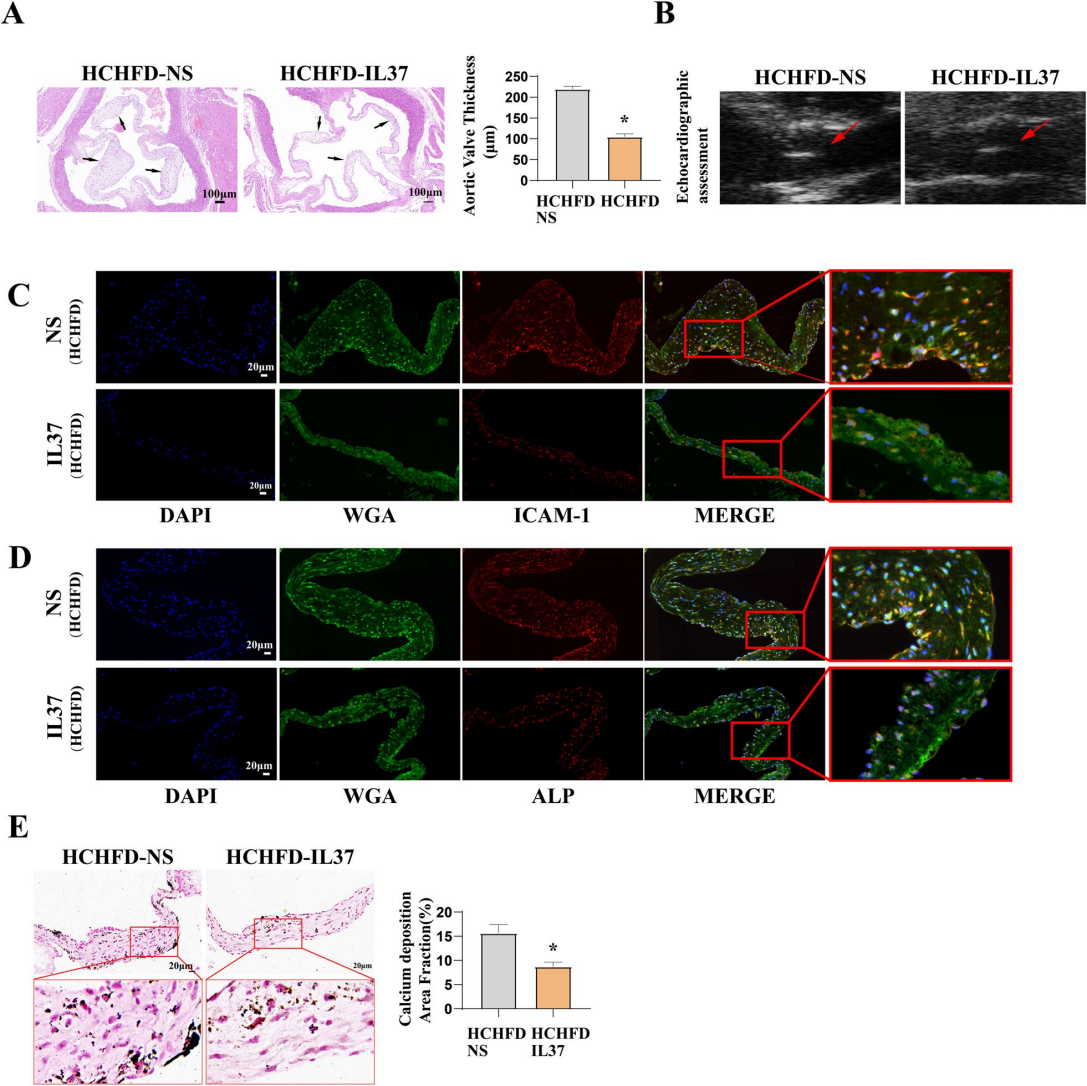
IL-37 treatment suppresses valvular inflammatory and osteogenic responses caused by HCHFD in *Ldlr*^*−/−*^ hamsters. **A** Representative histology images show that IL-37 attenuates aortic valve thickening in hamsters receiving prolonged HCHFD (bar = 100 µm). **B** Echocardiographic assessment showed that IL-37 attenuated HCHFD-induced aortic valve thickening in hamsters. **C** and **D** Representative immunofluorescence images show that IL-37 reduces the expression of ICAM-1 and ALP mediated by HCHFD in hamsters (bar = 20 µm). **E** Echocardiographic assessment showed that IL-37 attenuated calcium deposition (black calcium particles) in hamsters receiving prolonged HCHFD (bar = 20 µm). HCHFD + NS group n = 9. HCHFD + IL-37 group n = 7. *P < 0.05. vs. HCHFD-NS group. The data are presented as the means ± SEM. NS, normal saline; other abbreviations as in Fig. 2

IL-37 is an immunomodulatory member of the IL-1 family that broadly inhibits innate and acquired immune responses in vitro and in vivo. (Boraschi et al. 2011; Dinarello et al. 2016) As presented in Fig. 7A and B, IL-37 markedly reduced aortic valve thickness in *Ldlr*^*−/−*^ hamsters. Echocardiographic assessment revealed that IL-37 treatment could upregulate aortic valve orifice area (AVA) and later reduce transvalvular pressure (Supplemental Table S4). To evaluate the effect of IL-37 in vivo, we examined the expression of inflammatory and osteogenic biomarkers in aortic valve in *Ldlr*^*−/−*^ hamsters. Consistent with our in *vitro* observations, we found that IL-37 markedly reduced the expression of ICAM-1 and ALP (Fig. 7C and D) and calcium deposition (Fig. 7E). These data confirmed that IL-37 suppresses valvular pro-inflammatory and osteogenic activity in vivo and indicated that anti-inflammatory approaches may have therapeutic potential for suppression of AGE-LDL induced valvular osteogenic activity.

## Discussion

We report the following novel findings: (1) Patients with CAVD exhibit higher levels of AGE-LDL in serum compared to the non-CAVD group; (2) Prolonged exposure to a high-cholesterol, high-fat diet leads to increased circulating AGE-LDL levels and accelerates the progression of aortic valve calcification in vivo; (3) AGE-LDL induces inflammatory and osteogenic responses, and this effect on HAVICs is mediated by RAGE and involves the activation of the NF-κB signaling; (4) IL-37 and H3k27me3 Regulation: Patients with CAVD exhibit lower endogenous expression of IL-37 in aortic valve interstitial cells. This reduced expression is associated with increased levels of H3k27me3 in response to AGE-LDL stimulation and; (5) In hamsters, IL-37 treatment attenuates aortic valve thickening and reduces inflammatory and osteogenic response following the exposure to HCHFD.

Numerous clinical studies have demonstrated an association between circulating AGE-LDL and the risk of metabolic diseases (Hunt et al. 2013; Lopes-Virella et al. 2011). Recently, it was discovered that AGE-LDL causes atherosclerosis and inflammation (Reaves et al. 2000). Additionally, there are similarities in the pathophysiology and risk factors between CAVD and atherosclerosis. It is well recognized that aortic valve inflammation accelerates the development of CAVD (Eva Sikura et al. 2021). However, the role of AGE-LDL in CAVD remains unclear. Our present study demonstrates that increased AGE-LDL levels are found to be a risk factor.

AGE-LDL is formed when LDL cholesterol undergoes glycation, a non-enzymatic reaction where sugar molecules bind to proteins. RAGE is a cell surface receptor that binds to AGEs, including AGE-LDL. The binding of AGE-LDL to RAGE on valvular interstitial cells triggers a series of inflammatory responses. This interaction activates NF-κB signaling pathways, leading to the expression of pro-inflammatory and osteogenic proteins (Cote et al. 2013). The AGE-RAGE axis is implicated in the pathophysiology of atherosclerosis, which shares common mechanisms with CAVD (Wang et al. 2022). Research indicates that blocking the RAGE pathway can attenuate the progression of aortic valve calcification, suggesting a therapeutic target for CAVD (Deng et al. 2020). Studying the molecular mechanisms helps to better understand the role of AGE-LDL/RAGE interactions in the calcification process of CAVD.

Although previous research has shown that AGE-LDL is known to induce foam cell formation and promotes inflammatory response (Cheng et al. 2013; Deng et al. 2020), it remains unknown whether AGE-LDL induces inflammatory and osteogenic response in HAVICs. The underlying mechanisms are also unclear and deserve further exploration. ALP is early osteoblastic differentiation biomarker in mineral deposition (Zeng et al. 2017; Piperi et al. 2015). Previous studies reported that chronic activation of RAGE by exogenous AGEs contributes to the upregulation and activation of NF-κB in neuroinflammation, cardiac fibroblasts, macrophages and neuroblastoma cells (Bierhaus et al. 2001; Wang et al. 2013; Sanchez-Fernandez et al. 2021; Liu et al. 2019). We also demonstrate that AGE-LDL-induced production of ICAM-1, IL-6 and ALP by HAVICs is closely associated with the RAGE overexpression. In addition, RAGE silencing downregulated inflammatory and osteogenic response by partly modulating NF-κB signaling. AGE-LDL induced the expression of ICAM-1, IL-6 and ALP appears to be associated with the NF-κB signaling since treatment with a specific inhibitor of NF-κB signaling attenuated the upregulation of ICAM-1, IL-6 and ALP expression following AGE-LDL stimulation. Our results first demonstrated that AGE-LDL has a profound effect on NF-κB phosphorylation in HAVICs. The effect of RAGE inhibition on NF-κB phosphorylation and the effect of NF-κB inhibition on ICAM-1, IL-6 and ALP expression indicated that AGE-LDL upregulated expression of inflammatory cytokines and osteogenic production through RAGE/NF-κB signaling.

Here, we observed a significant increase in serum AGE-LDL levels after 4 weeks of HCHF diet without obvious aortic valve stenosis. No significant aortic stenosis was observed until 16 weeks of continuous feeding. Therefore, we developed a hamster model with calcified aortic valve after a HCHF diet for 16 weeks to investigate the role of AGE-LDL on the progression the progression of aortic valve calcification. More importantly, given 16 weeks’ of HCHF diet on hamster can lead to a significant increase in circulating AGE-LDL levels and stenosis of the aortic valve which is characterized by the aggregation of inflammatory and osteogenic products on valvular surface. Based on these findings, we found that elevated circulating AGE-LDL potentially contribute to aortic valve calcification.

The vitro and vivo experiments have demonstrated that IL-37 significantly reduces the inflammatory response and also has multiple effects on cell metabolism in addition to suppressing inflammatory cytokines in the body (Cavalli et al. 2017; Hakki et al. 2018; Tang et al. 2016). The present study found that AGE-LDL enhanced the level of H3k27me3 on the promoter of IL-37, which may provide a novel mechanism underlying the decrease of endogenous IL-37 in CAVD. Moreover, the exogenous IL-37 inhibited the AGE-LDL induced inflammatory and osteogenic response and attenuated valvular leaflet thickness.

Calcification in aortic valve appears first as microcalcifications, then merged to larger calcifications that accompanied by severe inflammatory reaction. Von Kossa stringing is a common calcium material, which is often used to evaluate the mineralization ability of cement oblasts, periodontal ligament cells and osteoblasts (Weiss et al. 2006; Lu et al. 2022). Many studies have confirmed that von Kossa staining is used to assess the ability of AVIC to produce calcium nodules by staining for calcium salts (Galeone et al. 2013). Von Kossa staining of the aortic valve in our study also demonstrated that abundant mineralization in calcified area but not in control group.

Here are some limitations in this study. First, the potential effect of AGE-LDL induced on the expression of osteogenic mediators, such as RUNX and BMP need to be evaluated in future studies. Second, primary cells isolated from human aortic valves and cultured in vitro may behave differently than they do in vivo, potentially affecting the study’s relevance to clinical conditions. Third, while our findings provide valuable insights into aortic valve calcification, future studies could explore the interplay between atherosclerosis and valve calcification.

In conclusion, AGE-LDL promotes inflammatory and osteogenic responses via AGE-LDL/RAGE/NF-ĸB in HAVICs and can be suppressed by IL-37 both in vitro and vivo. These findings underscore the potential therapeutic significance of targeting the AGE-LDL/RAGE/NF-ĸB axis as a novel strategy for managing calcific aortic valve disease.

### Supplementary Information

Supplementary material.

## Data Availability

All data generated or analysed during this study are included in this published article and its supplementary information files.

## Abbreviations

ALP: Alkaline phosphatase
AVS: Aortic valve stenosis
AGE-LDL: Advanced glycation end product-modified low-density lipoprotein
AGEs: Advanced glycation end-products
CAVD: Calcific aortic valve disease
HCHFD: High-carbohydrate and high-fat diet
HAVICs: Human aortic valve interstitial cells
ICAM-1: Intercellular cell adhesion molecule-1
IL-6: Interleukin-6
IL-37: Intherleukin-37
Ldlr^−^^/^^−^: Low-density lipoprotein receptor deletion
NF-κB: Nuclear factor kappa-B
RAGE: Receptor for advanced glycation end product
