# Supplementary material for: Advanced glycation end product-modified low-density lipoprotein promotes pro-osteogenic reprogramming via RAGE/NF-κB pathway and exaggerates aortic valve calcification in hamsters
Source: Mol Med. 2024 Jun 5;30:76. doi: 10.1186/s10020-024-00833-8 (PMC11155186; doi:10.1186/s10020-024-00833-8)
Supplement: Supplementary file 1 — Supplementary material. [file 10020_2024_833_MOESM1_ESM.docx]

**SUPPLEMENTAL DATA FOR**

**Advanced Glycation End Product-modified Low-density Lipoprotein Promotes Pro-osteogenic Reprogramming via RAGE/NF-κB Pathway and Exaggerates Aortic Valve Calcification in Hamsters**

Xi Yang^1,2,3^, Jingxin Zeng^1,2,3^, Kaiji Xie^1,2,3^, Shuwen Su1^,2,3^, Yuyang Guo^1,2,3^, Hao Zhang^1,2,3^, Jun Chen^1,2,3^, Zhuang Ma^1,2,3^, Zezhou Xiao^4^, Peng Zhu^4^, Shaoyi Zheng^4^, Dingli Xu1^,2,3*^, Qingchun Zeng^1,2,3*^

1. State Key Laboratory of Organ Failure Research, Department of Cardiology, Nanfang Hospital, Southern Medical University, 510515, Guangzhou, China
2. Guangdong Provincial Key Laboratory of Shock and Microcirculation, Southern Medical University, 510515, Guangzhou, China
3. Bioland Laboratory (Guangzhou Regenerative Medicine and Health Guangdong Laboratory), 510005, Guangzhou, China
4. Department of Cardiovascular Surgery, Nanfang Hospital, Southern Medical University, 510515, Guangzhou, China

***Corresponding author**:

Dingli Xu, MD **E-mail：**[dlxugz@163.com](mailto:dinglixu@fimmu.com).

Qingchun Zeng, M.D. **E-mail：**[qingchunzeng@smu.edu.cn](mailto:qingchunzeng@smu.edu.cn).

Tel.: +86-020-61641493. Fax: +86-020-61360416.

Key Laboratory for Organ Failure Research, Department of Cardiology, Nanfang Hospital, Southern Medical University, 1838 Northern Guangzhou Ave, Guangzhou, 510515, China.

**Supplemental Figure 1**

Inclusion flow diagram for our study

November 2018 to December 2019

hospitalized patients in Cardiology department

patients treated with antibiotics or probiotics within one month were excluded

Informed consent

N = 582

Fasting blood samples

Clinical data collection

Excluded (n=226)

Not meeting inclusion criteria (n= 156)

Clinical data missing (n= 21)

blood samples not collected (n=49)

N = 356

**Supplemental Figure 2**


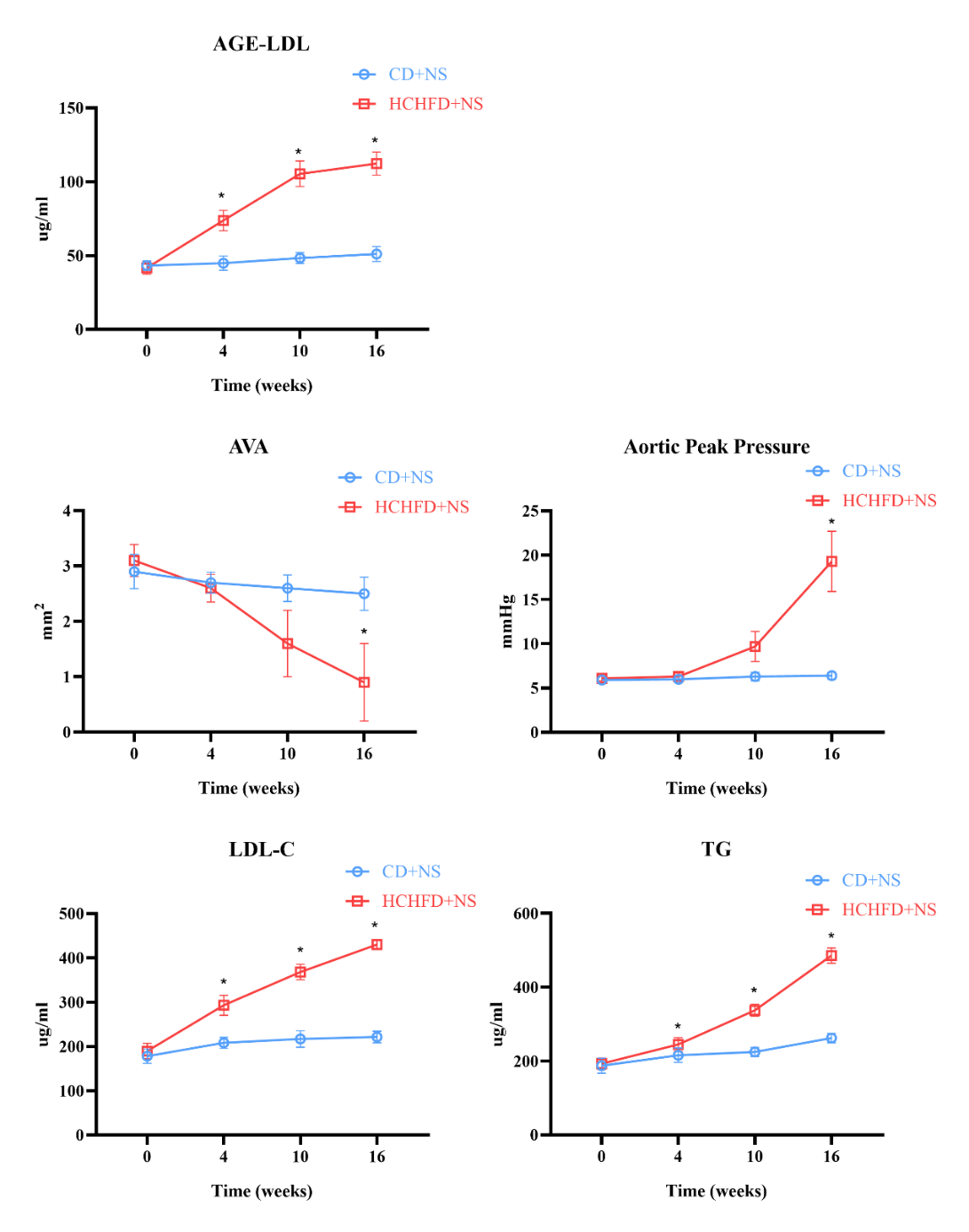
Summary of hamsters fed chow diet or HCHFD


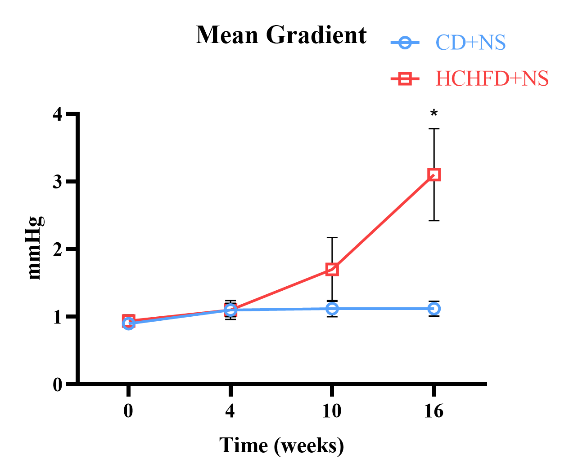

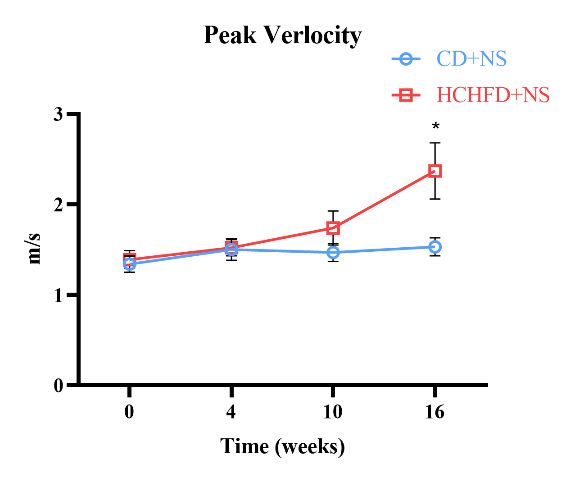


Data are presented as mean ± SEM.

* *P* < 0.05. HCHFD + NS vs. CD + NS at corresponding time points.

Abbreviations: CD, chow diet; HCHFD, high fat/high carbohydrate diet; AVA, aortic valve orifice area; AGE-LDL, advanced glycation end products modified low density lipoproteins; TG, triglyceride; LDL-C, low-density lipoprotein cholesterol.

**Supplemental Table 1**

Comparison of patient characteristics between non-CAVD and CAVD group.

| Variable | Non-CAVD (n=275) | CAVD (n=81) | *P* Value |
| --- | --- | --- | --- |
| Age, years | 65.8 ± 7.7 | 70.4 ± 7.2^*^ | < 0.001 |
| Male sex, No. (%) | 178 (65.0) | 58 (71.6) | 0.266 |
| LDL-C, mmol/l | 2.5 ± 0.5 | 2.9 ± 0.6^*^ | < 0.001 |
| TG, mmol/l | 1.5 ± 0.4 | 1.6 ± 0.5 | 0.052 |
| UA, mg/dl | 383.9 ± 135.2 | 406.7 ± 124.6 | 0.176 |
| CR, µmol/l | 79.1 ± 25.7 | 80.6 ± 25.7 | 0.636 |
| AVA, cm^2^ | 3.28 ± 1.21 | 0.73 ± 0.19 | < 0.001 |
| Mean gradient, mmHg |  | 大于40 | N |
| AGE-LDL, ug/ml | 17.9 ± 4.9 | 59.9 ± 11.2^*^ | < 0.001 |
| HbA1c | 5.2 ± 0.5 | 7.8 ± 0.9^*^ | < 0.001 |
| Diabetes, No. (%) | 69 (25.2) | 30 (37.0)^*^ | 0.037 |
| Hypertension, No. (%) | 156 (56.9) | 54 (66.7) | 0.117 |
| Coronary artery disease, No. (%) | 110 (40.1) | 37 (45.7) | 0.374 |
| Heart failure, No. (%) | 28 (10.2) | 35 (43.2)^*^ | < 0.001 |

*P* values were obtained by an unpaired t-test, or chi-squared test.

* *P* < 0.05. CAVD vs. non-CAVD.

^a^ Data are presented as mean ± SD or median (interquartile range), categorical variables are presented as %.

Abbreviations: TG, triglyceride; LDL-C, low-density lipoprotein cholesterol; UA, Uric acid; CR, creatinine; AVA, aortic valve orifice area; AGE-LDL, advanced glycation end-products modified low density lipoprotein.

**Supplemental Table 2**

Logistic regression analyses of plasma AGE-LDL levels for CAVD model.

| Model | Odds Ratio (95% CI) | *P* Value |
| --- | --- | --- |
| Unadjusted | 1.26 (1.18-1.34) | ＜0.001 |
| Model 1 | 1.26 (1.18-1.34) | ＜0.001 |
| Model 2 | 1.30 (1.18-1.43) | ＜0.001 |
| Model 3 | 1.41 (1.20-1.66) | < 0.001 |

Odds ratio shown were for plasma AGE-LDL level as a continuous variable.

Model 1 adjusted for age and sex.

Model 2 adjusted for all factors in model1 plus LDL-C, TG, UA, HbA1c, AVA,

Mean gradient and CR.

Model 3 adjusted for all factors in model 2 plus hypertension, diabetes mellitus, coronary artery disease and heart failure.

**Supplemental Table 3**

Comparison of CAVD patient characteristics between diabetes and non-diabetes group.

| Variable | Non-diabetes (n=35) | Diabetes (n=46) | *P* Value |
| --- | --- | --- | --- |
| Age, years | 69.4 ± 6.8 | 71.2 ± 7.5 | 0.273 |
| Male sex, No. (%) | 27 (46.6) | 31 (53.5) | 0.335 |
| LDL-C, mmol/l | 2.8 ± 0.6 | 3.0 ± 0.6 | 0.142 |
| TG, mmol/l | 1.5 ± 0.4 | 1.7 ± 0.6 | 0.086 |
| UA, mg/dl | 389.9 ± 118.8 | 419.5 ± 128.7 | 0.292 |
| CR, µmol/l | 79.5 ± 25.8 | 80.4 ± 26.5 | 0.887 |
| AGE-LDL, ug/ml | 58.6 ± 8.9 | 60.9 ± 12.6 | 0.353 |
| HbA1c | 4.9 ± 0.5 | 6.7 ± 1.5^*^ | < 0.001 |
| Hypertension, No. (%) | 22 (40.7) | 32 (59.3) | 0.464 |
| Coronary artery disease, No. (%) | 17 (45.9) | 20 (54.1) | 0.660 |
| Heart failure, No. (%) | 14 (40.0) | 21 (60.0) | 0.821 |

*P* values were obtained by an unpaired t-test, or chi-squared test.

* *P* < 0.05. Diabetes vs. Non-diabetes.

^a^ Data are presented as mean ± SD or median (interquartile range), categorical variables are presented as %.

Abbreviations: TG, triglyceride; LDL-C, low-density lipoprotein cholesterol; UA, Uric acid; CR, creatinine; AGE-LDL, advanced glycation end-products modified low density lipoprotein.

**Supplemental Table 4**

Summary of hamsters pumped saline or IL-37

| Variable | HCHFD+NS  (n=9) | HCHFD+IL-37  (n=7) | *P* value |
| --- | --- | --- | --- |
| Aortic Peak Pressure (mmHg) | 18.7 ± 1.6 | 11.4 ± 1.1^*^ | 0.003 |
| AVA (mm^2^) | 0.8 ± 0.1 | 1.7 ± 0.3^*^ | 0.007 |
| AGE-LDL (ug/ml) | 117.3 ± 2.4 | 109.7 ± 5.5 | 0.191 |
| TG (mg/dl) | 473.2 ± 8.9 | 461.2 ± 15.2 | 0.485 |
| LDL-C (mg/dl) | 434.6 ± 7.9 | 417.6 ± 9.6 | 0.189 |

Data are presented as mean ± SEM.

* *P* < 0.05. HCHFD + IL-37 vs. HCHFD + NS.

Abbreviations: NS, normal saline; IL-37, interleukin 37; HCHFD, high fat/high carbohydrate diet; AVA, aortic valve orifice area; AGE-LDL, advanced glycation end products modified low density lipoproteins; TG, triglyceride; LDL-C, low-density lipoprotein cholesterol.

**Supplemental Table 5**

**Characteristics of Non-CAVD and CAVD patients for aortic valve sample study**

Comparison of patient characteristics between non-CAVD and CAVD group.

| Variable | Non-CAVD (n=6) | CAVD (n=6) | *P* Value |
| --- | --- | --- | --- |
| Age, years | 60.0 ± 2.2 | 71.0 ± 2.1 ^*^ | < 0.001 |
| Male sex | 3 | 3 | NS |
| LDL-C, mmol/l | 2.2 ± 0.3 | 3.4 ± 0.7^*^ | < 0.001 |
| TG, mmol/l | 1.3 ± 0.2 | 1.5 ± 0.5 | NS |
| UA, mg/dl | 383.1 ± 130.2 | 403.7 ± 121.6 | NS |
| CR, µmol/l | 75.1 ± 20.7 | 77.2 ± 21.8 | NS |
| Glucose, mmol/L | 5.1± 1.8 | 11.6± 3.5^*^ | < 0.001 |
| AVA, cm^2^ | 3.18 ± 1.2 | 0.63 ± 0.2^*^ | < 0.001 |
| Mean gradient, mmHg | 17 ± 2.4 | 40 ± 3.2^*^ | < 0.001 |
| AGE-LDL, ug/ml | 16.6±4.2 | 59.2 ± 10.1^*^ | < 0.001 |
| HbA1c | 4.7 ± 0.3 | 7.0 ± 0.8^*^ | < 0.001 |

*P* values were obtained by an unpaired t-test, or chi-squared test.

* *P* < 0.05. CAVD vs. non-CAVD.

^a^ Data are presented as mean ± SD or median (interquartile range), categorical variables are presented as %.

Abbreviations: TG, triglyceride; LDL-C, low-density lipoprotein cholesterol; UA, Uric acid; CR, creatinine; AVA, aortic valve orifice area; AGE-LDL, advanced glycation end-products modified low density lipoprotein.

**CHIP-qPCR**

a. The cells were collected by centrifugation and 1% formaldehyde was added to cross-link them for 10 min. Then glycine solution was added to neutralize the formaldehyde for 5 min. The cells were washed with PBS and the precipitate was collected by centrifugation and stored at -80 ℃ for subsequent experiments.

b.1mL Lysis Buffer 110μL Protease Inhibitor and 10 μL DTT were added into the cell precipitate, which was mixed for 5 min at 4 °C. 1mL Lysis Buffer 210 μL Protease Inhibitor and 10μL DTT were added to resuspend the cells and mixed for 10 min. Then 1.7 mL Lysis Buffer 317 μL Protease Inhibitor and 17 μL DTT were added to resuspend the precipitate and lysed on ice for 10 min.

c. Cell precipitates were lysed in ice water at 4 °C using a non-contact ultrasonic cell breaker and 150 μL of Agarose beads were mixed with the supernatant, the supernatant was removed after centrifugation.

d. After mixing 1mL Block Solution with 40μL protein A/G-beads, the magnetic beads were mixed with 40μL Lysis Buffer and divided equally into two portions.

e. The pre-washed samples were divided into 3 portions: 100 μL (input), 800 μL (IgG) and 800 μL (IP). Add 0.3mL 1×Elution Buffer to the samples in the Input group and store at -20 ℃. Add the ChIP primary antibody to the samples in the IP group. Add an equal amount of IgG antibody to the IgG sample group and incubate at 4 °C for 12 to 16 hours. The prepared protein A/G-beads were added to the IP group and IgG group respectively and incubated for 30 min at room temperature on a vertical mixer.

f. Wash and collect magnetic beads in IgG and IP group samples. After adding 200 μL of Elution Buffer to the IgG and IP samples respectively, and incubated at 65 ℃ for 15 min. Then the supernatant was transferred to a new centrifuge tube.

g. Take out the Input group and incubate with the eluted IgG and IP group samples at 65 ℃ for 6 hours to perform reverse cross-linking. Add 0.2mL 1×TE and 8μL RNaseA to each group and incubate at 37 ℃ for 30~120 minutes. Then add 4μL EDTA and 4μL Proteinase K to each group and incubate at 55 ℃ for 120 minutes.

h. A mixture of phenol, chloroform and isoamyl alcohol was added to each group, and the supernatant was retained by centrifugation. Sodium chloride solution, Glycogen and anhydrous ethanol were added to the supernatant and placed at -20 ℃ for 30~120 minutes.

i. The precipitate was washed with 80% ethanol, and after the precipitate was completely air-dried, enzyme-free double-distilled water was added to dissolve the DNA, and the resulting DNA solution was used for qPCR experiments to verify the enrichment efficiency.

**Supplemental Figure 3**

The extent of LDL protein glycation was evaluated using spectrofluorometric measurements of pentosidine formation (with an excitation wavelength of 335 nm and an emission wavelength of 385 nm). The results showed that the fluorescence density of AGE-LDL was significantly increased compared to the control group BSA (bovine serum albumin), indicating the presence of a large amount of glycosylation products in low-density lipoprotein.

The data are presented as the means ± SD.

* *P* < 0.05. AGE-LDL vs. BSA.

**Supplemental Figure 4**


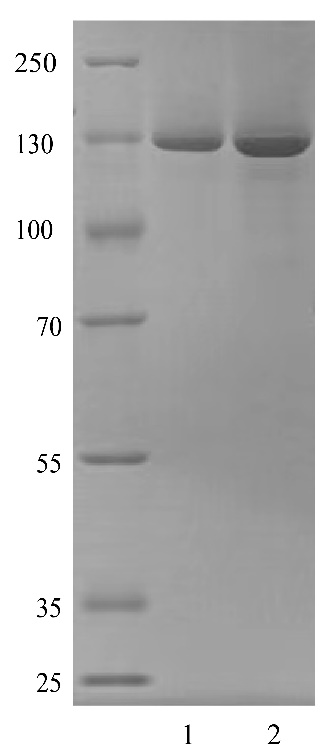


1: various oxidatively modified LDLs include ox-LDL, AGE-LDL and MDA-LDL

2: purified AGE-LDL

We used purified AGE-LDL and suspension of various oxidatively modified LDLs include ox-LDL, AGE-LDL and MDA-LDL to test the specificity of AGE-LDL antibodies by Immunoblotting. The images showed only one band that specifically bound to AGE-LDL in both the suspension of various oxidatively modified LDLs and the purified AGE-LDL, indicating the specificity of AGE-LDL antibodies.
